# Ultrasound measurement of optic nerve sheath diameter in a healthy adult Colombian population

**DOI:** 10.1186/s12883-023-03062-4

**Published:** 2023-01-13

**Authors:** Guillermo Trocha, Andrés Bonilla, Camilo Romero, Jonathan Palacios, Nicolas Molano-Gonzalez

**Affiliations:** 1 Department of Neurology, Fundación Cardioinfantil-Instituto de Cardiología, Bogotá, Colombia; 2 Department of Critical Care Medicine, Fundación Cardioinfantil-Instituto de Cardiología, Bogotá, Colombia; 3 Clinical Research Group, School of Medicine and Health Science, Universidad del Rosario, Bogotá, Colombia

**Keywords:** Optic nerve sheath diameter, Ultrasonography, Healthy Adults, Latin America

## Abstract

**Background:**

Measurement of the optic nerve sheath diameter (ONSD) provides a rapid, safe, and easy method for detecting increased intracranial pressure (ICP). However, the normal mean and upper limit values may vary according to sex, age, ethnicity, and ultrasound technique.

**Aim:**

We aimed to obtain the mean ONSD in a healthy Colombian adult population and to correlate it with demographic and anthropometric measures.

**Methods:**

In a prospective study using a 10–13 MHz linear ultrasound probe, eye transverse diameter (ETD) and ONSD in the transverse (ONSD-TP) and sagittal planes (ONSD-SP) were measured in healthy adult volunteers in Bogota, Colombia.

**Results:**

A total of 100 healthy subjects were included, with a mean age of 26,7 ± 8,3 years and 62 women. The mean ETD, ONSD-TP and ONSD-SP was 23.11 mm (95% confidence interval (CI): 22.90 mm-23.32 mm), 3.96 mm (95% CI: 3.85 mm-4.07 mm) and 4.0 mm (95% CI: 3.90 mm-4.11 mm), respectively. The ONSD in both planes ranged from 2.35 mm to 5.20 mm. There was a significant correlation between ONSD-SP and ONSD-TP (*p* < 0.0001) but no correlation between the ocular measures and demographic or anthropometric variables (*p* > 0.05). The intraclass correlation between the eyes was statistically significant.

**Conclusion:**

Our study shows that ultrasound-measured ONSD in healthy adults in Colombia is similar to that found worldwide. An ONSD of 5.5 mm may be considered the upper limit for healthy adults in Colombia. ONSD can be measured in either plane; there is a good correlation between the two eyes; and ONSD is not modified by demographic or anthropometric characteristics.

## Background

Increased intracranial pressure (ICP) is a common complication of many neurological and systemic disorders. Its presence leads to unfavourable visual and neurological outcomes, and it is associated with an increased risk of mortality [1]. Early detection, monitoring and treatment of ICP are associated with better outcomes and decreased mortality [2]. Currently, a variety of invasive and noninvasive techniques to diagnose and monitor ICP are being implemented. However, invasive methods require highly qualified personnel, and expensive equipment and carry a risk of serious complications such as infection, brain haemorrhage and catheter malfunction, which occur in 6–32.8% of subjects [3].

Among the noninvasive methods, ultrasound (US) measurement of the optic nerve sheath diameter (US-ONSD) provides a faster and safer alternative that is easy to perform and offers the opportunity for real-time assessment. This measurement has a good correlation with papilledema and measured intracranial pressure [4, 5]. As ICP increases, the subarachnoid space around the optic nerve expands, and on US, protrusion of the optic nerve head and increased ONSD become evident.

Nevertheless, the diagnosis of increased ICP by US-measured ONSD requires knowledge of the normal parameters and cut-off values. Normative means and upper limits for the US-ONSD have been established in different healthy populations. US-ONSD measurements are taken at 3 mm behind the posterior globe margin, from the inner edge to the inner edge of the optic nerve sheath. However, considering that the length and size of the eyeball could change in cases of myopia, hypermetropia, congenital or acquired glaucoma and some other disorders that might influence the accuracy of US-ONSD measurements, the ratio between ONSD and the eye transverse diameter (ETD) has been proposed to improve the reliability for ICP detection [6]. In healthy adults, ONSD measurements of up to 5 mm have been considered normal. Measurements > 5 mm (bilaterally) are correlated with ICP above 20 mmHg, with a linear association up to 7.5 mm, at which the diameter appears to plateau. However, these values vary according to sex, age, ethnicity and US operator [7, 8]. Normative values for the ONSD/ETD ratio have been published less frequently.

To the best of our knowledge, there is scarce information on normal values of ONSD in healthy adults from Colombia and Latin America [9]. The aim of this study was to obtain the normative values for US-measured ONSD (transverse and sagittal planes) and for the ONSD/ETD ratio in healthy adult volunteers. We also aimed to correlate these values with the demographic and anthropometric characteristics of the study subjects.

## Materials and methods

A cross-sectional descriptive study was carried out between January 2021 and July 2021 at *Fundacion Cardioinfantil* in Bogotá, Colombia. Due to the current pandemic, biosafety measures as required by the hospital and local and national authorities were followed in all procedures.

This study was approved by the institutional research ethics committee (Approval number: CEIC-4472–2021) and prior written informed consent was provided by all included subjects.

### Participants

Healthy adult volunteers aged 18 years and older, with no past or current neurological or neuro-ophthalmological diseases and normal neurological exams were included. Subjects with a disease, condition or treatment that could interfere with or alter, as judged by the investigators, the US measurements or intracranial pressure were excluded. The study subjects consisted mainly of medical students and hospital workers.

For all included subjects the following data were obtained: sex, age, height, weight, body mass index [BMI], head circumference [HC]), blood pressure, heart rate, body temperature, current symptoms, COVID-19 symptoms, and past medical history. A detailed direct bilateral fundoscopy was performed for each of the studied subjects. Their height was measured in metres with a standard wall metre scale, their weight was measured in kilograms with a calibrated office electronic scale, and their HC was measured with a standard nonelastic metre at the widest possible occipitofrontal circumference.

### Optic nerves heath ultrasound

**Fig. 1:**
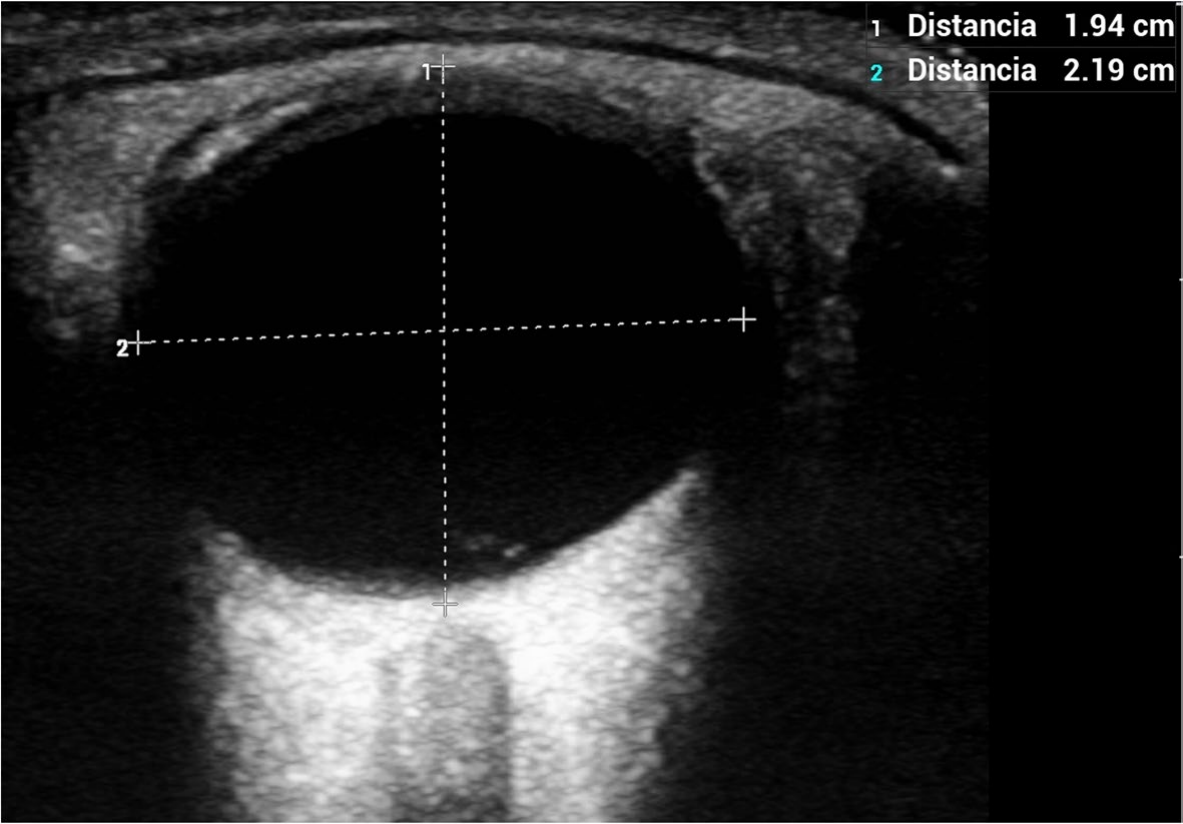
The ETD was measured in the section showing the maximal transverse diameter of the eyeball

**Fig. 2:**
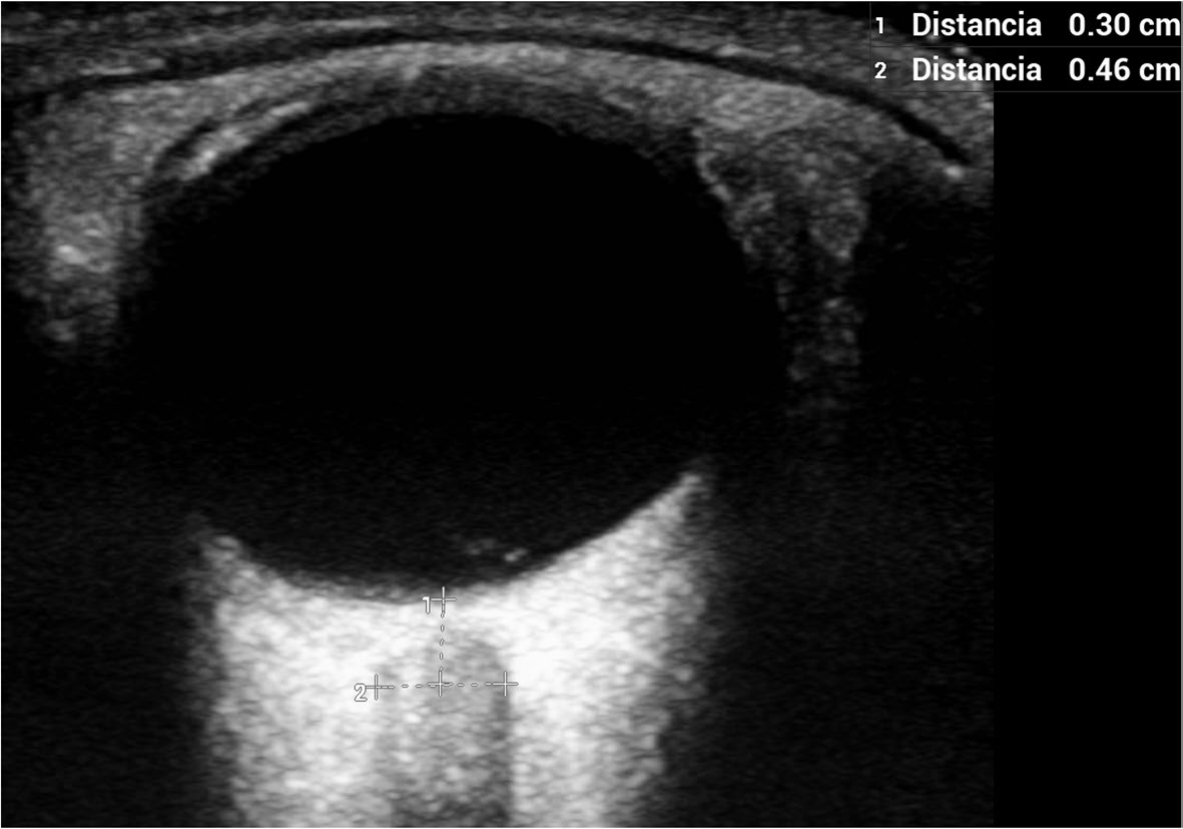
The ONSD was measured 3 mm behind the papilla from the inner edge to the inner edge of the optic nerve sheath surrounding the optic nerve

US-ONSD measurements were carried out by one intensivist and three neurologists using a portable Edge Sonosite Fujifilm ultrasound system. A standard orbital bidimensional (2D) imaging mode with a 10–13 MHz linear US probe at maximal gain, 6 cm of depth and 0 on the greyscale was used. The system’s error in the B mode in the interval between 0–26 cm was <  ± 2% for axial, lateral and diagonal distances. All ocular measurements were performed with the subjects in the supine position with the head at 30 degrees elevation. The probe was applied to the upper closed eyelids with coupling gel, in the superolateral margin of the orbit and with the eyes in primary gaze. This was done to both become aligned with the direction of the nerve and to prevent excessive pressure from being exerted on the eye. The position of the probe was adjusted to clearly display the entry of the optic nerve into the globe. The right eye was assessed first, and the maximal ETD was measured (Fig. 1) followed by ONSD in the transverse plane (ONSD-TP) at 3 mm behind the posterior globe margin. The probe was then rotated 90 degrees and the ONSD in the sagittal plane (ONSD-SP) was measured at the same distance. The ONSD was measured from the inner edge to the inner edge of the optic nerve sheath (Fig. 2). The procedure was repeated for the left eye. Two measurements were taken at each position in both eyes. The mean of each measurement was calculated to minimize intraobserver variability. ONSD-TP was used to calculate the ONSD/ETD ratio, assuming the same position of the probe for both measurements.

### Statistical analysis

Quantitative variables are reported as the mean and standard deviation, whereas qualitative variables are reported as absolute and relative frequencies. To estimate the mean of the ETD, ONSD-TP, ONSD-SP and ONSD/ETD across both eyes, we used random-effects models to account for the correlation of the measures from both eyes in a given patient. From this, we were able to obtain an adjusted mean estimate for the parameter of interest as well as the intraclass correlation coefficient. In further analysis, we aimed to evaluate marginal associations between the physiologic characteristics and ocular parameters, which was done with mixed-linear models. All statistical analyses were carried out in R (v. 4.1.1).

## Results

A final sample of 100 healthy volunteers was obtained, with a mean age of 26.7 ± 8.3 years, 62 of whom were female. The mean weight, height, BMI, and HC were 65.5 ± 12.5 kg, 167.4 ± 9.9 cm, 23.3 ± 3.4 kg/m^2^ and 56.0 ± 2.6 cm, respectively (Table 1).

**Table 1:** Demographic and anthropometric characteristics of the subjects (*n* = 100)

	**(n,%)/(mean ± SD)**	**Minimum**	**Maximum**
Sex-Male	38 (38%)		
Age (years)	26 ± 8	20	73
Weight (kg)	65,5 ± 12,5	42	96
Height (cm)	167,4 ± 9.9	145	196
BMI (kg/m2)	23.3 ± 3,4	17,4	38
HC (cm)	56,0 ± 2.6	51	68

*SD* Standard deviation, *BMI* Body mass index, *HC* Head circumference

The eye transverse diameter (ETD) measures ranged from 20.50 mm to 29.20 mm. The ONSD-TP measures ranged from 2.35 mm to 5.15 mm, and the ONSD-SP measures ranged from 2.60 mm to 5.20 mm. The means and 95% confidence intervals (95% CI) for ETD, ONSD-TP and ONSD-SP were 23.11 mm (95% CI: 22.91 mm-23.33 mm), 3.96 mm (95% CI: 3.85 mm-4.07 mm) and 4.0 mm (95% CI: 3.90 mm-4.11 mm), respectively. The intraclass correlation coefficients between eyes for the ETD, ONSD-TP, and ONSD-SP were of 0.73, 0.84 and 0.88, respectively. The ONSD/ETD ratio range from 0.1 to 0.23, with a mean ratio of 0.17 (Table 2).

**Table 2:** Descriptive statistics for ETD, ONSD-TP, ONSD-SP and ONSD/ETD ratio

		**Mean (95% CI)**	**Minimum**	**Maximum**
**ETD (mm)**	Right	23,10 (22.87–23.33)	21,05	29,2
	Left	23,13 (22.91–23.36)	20,50	26,75
	Overall	23.11 (22.91–23.33)	20.5	29.2
**ONSD-TP (mm)**	Right	3,93 (3.83–4.05)	2,35	4,95
	Left	3,99 (3.88–4.11)	2,65	5,15
	Overall	3.96 (3.85–4.07)	2.35	5.15
**ONSD-SP (mm)**	Right	4,01 (3.90–4.12)	2,85	5
	Left	4,00 (3.89–4.11)	2,6	5,2
	Overall	4.0 (3.90–4.11)	2.60	5.20
**ONSD/ETD ratio**	Right	0,17 (0.17–0.18)	0.10	0.15
	Left	0.17 (0.17–0.18)	0.11	0.23
	Overall	0,17 (0,16–0.17)	0.10	0.23

*CI* Confidence interval, *ETD* Eye transverse bulb, *ONSD-TP* Optic nerve sheath diameter-transverse plane, *ONSD-SP* Optic nerve sheath diameter-sagittal plane, *ONSD/ETD* Optic nerve sheath diameter/eye transverse diameter

There was a statistically significant correlation between ONSD-SP and ONSD-TP (*p* < 0.0001) but there was no association between the mean ETD, ONSD and ONSD/ETD and sex, age, weight, height, BMI, or HC (*p* > 0.05) (Table 3).

**Table 3:** Correlations between ETD, ONSD-TP, ONSD/ETD ratio and demographic and anthropometric characteristics

	**ETD**			**ONSD-TP**			**ONSD/ETD**		
	Beta	Std. Err. of Beta	*p*- value	Beta	Std. Err. of Beta	*p*- value	Beta	Std. Err. of Beta	*p*- value
**Sex-Male**	0,400	0,219	0,070	0,141	0,113	0,216	0,003	0,005	0,521
**Age (years)**	-0,006	0,219	0,665	-0,006	0,007	0,369	0,000	0,000	0,428
**Weight (kg)**	0,014	0,008	0,104	0,003	0,004	0,505	0,000	0,000	0,894
**Height (cm)**	0,025	0,011	0,019	0,006	0,006	0,265	0,000	0,000	0,703
**BMI (kg/m2)**	0,002	0,031	0,952	0,003	0,016	0,854	0,000	0,001	0,896
**HC (cm)**	0,061	0,040	0,131	0,034	0,021	0,103	0,001	0,001	0,259

*ETD* Eye transverse bulb, *ONSD-TP* Optic nerve sheath diameter-transverse plane, *ONSD/ETD* Optic nerve sheath diameter/eye transverse diameter, *BMI* Body mass index, *HC* Head circumference

## Discussion

In an adult healthy volunteer population in Bogota, Colombia, using orbital US we obtained a mean ONSD-TP of 3.96 mm (95% CI: 3.85 mm-4.07 mm) and a mean ONSD-TP of 4.0 mm (95% CI: 3.90 mm-4.11 mm). For the ONSD/ETD ratio, the mean was 0.17 (95% CI: 0.16–0.17). The ranges for ONSD-TP and ONSD-SP were from 2.35 mm to 5.15 mm and from 2.60 mm to 5.20 mm, respectively. The ONSD/ETD ratio ranged from 0.1 to 0.23. We found a significant correlation between ONSD-TP and ONSD-SP (*p* < 0.0001), and the intraclass correlation between eyes was statistically significant and comparable with previous reports [5, 10]. We did not find any significant association between sex, age, BMI, HC and US measures of ONSD or ONSD/ETD (*p* > 0.05), which is consistent with the findings of a recent systematic review and meta-analysis and a previous review [9, 11]. Considering that the ONSD did not exceed 5.2 mm in the transversal or sagittal plane, a 30 degrees test to check for undetected intracranial hypertension was not necessary [12, 13].

Previous studies of US ONSD measures in healthy populations (children and adults) reported a range of 2.2 to 5.4 mm. In 67 subjects in the United Kingdom, the mean ONSD ranged between 2.4–4.7 mm (mean 3.2–3.6 mm) [14]. In 26 subjects in Greece, the range was between 2.2–4.9 mm (mean 3.6 mm), and in 136 subjects in Bangladesh, it ranged between 4.24–4.83 mm (mean 4.41 mm) [15, 16]. Higher values of US ONSD have been found in studies in China (range 4.7–5.4 mm, mean 5.1 mm) and Korea (range 4.6–5.2 mm, mean 4.9 mm) [10, 17]. US ONSD cut-off values for the diagnosis of increased ICP ranged from 4.1 to 5.7 mm, with a good correlation with invasive measures. Cut-off measures of US ONSD of 4.7 to 5.7 mm for an increased ICP diagnosis showed a sensitivity of 70–100% and specificity of 31.9–100% [18]. Normative values for ONSD/ETD in healthy subjects are seldom reported. Kim et al. presented the first study establishing a normal ONSD/ETD ratio by ultrasound in 585 healthy volunteers, reporting a mean of 0.18 (range 0.12–0.23), very similar to our results [11]. A US-ONSD/ETD ratio of 0.25 has been proposed as a threshold value with a sensitivity of 90% and specificity of 82.3% [6].

US ONSD measures have shown minimal interobserver and eye-to-eye variations. Interobserver variation ranges between 0.2–0.3 mm, and differences in measures between the axial and sagittal plane of US ONSD are in the range of 0–0.3 mm (mean 0.15 mm) [14]. Variability can also be minimized with appropriate training, and the learning curve for optimal orbital US-ONSD measurement is only between 10 and 25 scans [19]. The studies discussed above also found no association between US-ONSD and age, weight or height, further reducing the variability. A study including HC, did not find a relationship between US-ONSD and HC [16], although ONSD may vary by sex and ethnicity [8].

Although studies have reported on many parameters of US-ONSD in healthy populations, no global consensus exists on normal US-ONSD measures and cut-off values for the diagnosis of increased ICP [20]. There is also no universally accepted standardized protocol for sonographic assessment of the ONSD, even though proposals for protocols have been made [20]. Our study was carried out using the technique performed in most published studies.

Knowledge of the normal range of US ONSD in a healthy local population is essential to interpret the results of this diagnostic test. Based on the results of our study, prior research and expected deviations when measuring US-ONSD, we propose a normal range for US-ONSD of 2.35–5.20 mm in healthy adult Colombians healthy and a cut-off value for diagnosing increased ICP of 5.5 mm.

To the best of our knowledge, this is the first study to report normative values in a sample of exclusively Latin American subjects [9]. Relationships between demographic (sex, age) and anthropometric (weight, height, BMI, and HC) variables with US ONSD were analysed, and no association was found. The sample size was adequate and there was careful exclusion of subjects with potentially abnormal US-ONSD. The subjects were also examined in a systematic and standardized fashion by trained physicians.

This study has some limitations. First, we did not use neuroimaging to confirm the absence of disease/masses. However, patients with ICP typically present with papilledema, which is absent in less than 6% of subjects. In either case, certain symptoms were reported in both groups of patients with ICP (e.g., headache, visual field loss, diplopia), and they were the basis for our exclusion criteria [21]. Second, the mean age of our patients was 26.7 ± 8.3 years, which, considering the possible influence of ageing and brain atrophy on ONSD, would make the ranges found in this study narrower than they would be if older subjects had been included. However, previous studies have shown that ONSD remains similar throughout adult life and does not vary even in healthy adults aged 65 years or older [22]. Third, the interobserver variability was not analysed, as different investigators measured ONSD in different volunteers. A direct measurement of ICP was not performed, and how the upper values of US ONSD correlate with ICP could not be determined. Additionally, we did not compare the ONSD measurements with the results of other noninvasive methods.

We did not associate the standardized A-scan technique with the B-scan ocular ultrasound results. The latter could be affected by the so-called “blooming effect” making measurements unreliable as a recent review suggested [13, 23]. Finally, the lack of previous studies on healthy adult Latin American populations limits the possibility for regional comparisons. Despite the limitations mentioned and the knowledge that further research is needed on US ONSD and its correlation with increased ICP, our study helps build a reference of normative data for Latin American US measurements.

## Conclusion

US measurement of ONSD is a quick, noninvasive, and easy-to-perform bedside method to diagnose and monitor increased ICP. As there is no global consensus on the normal parameters of ONSD, US laboratories should strive to build normative data that allow clinicians to interpret the results of these scans. We consider that 5.5 mm should be used as the upper limit of normal US ONSD in Colombia. More research and training are needed for the US measurement of ONSD in Latin America.

## Data Availability

The datasets used and/or analysed during the current study are available from the corresponding author upon reasonable request.

## Abbreviations

ONSD: Optic nerve sheath diameter
ICP: Increased intracranial pressure
ETD: Eye transverse diameter
ONSD-TP: Optic nerve sheath diameter-Transverse plane
ONSD-SP: Optic nerve sheath diameter-Sagittal plane
US: Ultrasound
BMI: Body mass index
HC: Head circumference
